# Examining Older Adults’ Perspectives on the Built Environment and Correlates of Healthy Aging in an American Age-Friendly Community

**DOI:** 10.3390/ijerph17197056

**Published:** 2020-09-27

**Authors:** Kathy Black, Dylan J. Jester

**Affiliations:** 1 School of Aging Studies, College of Behavioral and Community Sciences, Sarasota-Manatee Campus, University of South Florida, Sarasota, FL 34243, USA; 2 School of Aging Studies, University of South Florida, Tampa, FL 33620, USA; djjester@usf.edu; 3 College of Public Health, University of South Florida, Tampa, FL 33620, USA

**Keywords:** active aging in place, environmental design, healthy aging, livable communities

## Abstract

Population aging has led to an increased focus on the environmental context in which we age. While researchers have identified significant health benefits associated with built community features such as housing, transportation and outdoor spaces and buildings, less attention has focused on the correlates of healthy aging and other characteristics via the perspective of community-dwelling older adults. This study utilized cluster analysis to examine health-related subgroups of older adults (*n* = 598) in an age-friendly community located in the United States, of which nearly half of its residents are age 60 and older. Linear regression was used to associate the health clusters with perceptions of built environmental features and socio-demographics. Four distinct profiles were identified, with the greatest preference for housing and transportation found among those reporting poorer health compared to those reporting excellent health across multi-dimensional healthy aging measures. Perceptions on the importance of built environmental features were also found to vary by age, income and home accessibility status. Findings suggest that older adults’ perceptions about built environmental features differ across health and home status as well as age and income, underscoring opportunities for public health action to better reach and engage older adults by life-course trajectories in age-friendly communities.

## 1. Introduction

Population aging impacts all facets of society including the built environment. As people age, a myriad of circumstances can affect their ability to manage at home. Moreover, normative age-related changes and pathological decline in older adults’ mobility can affect their access to necessary goods and services and may restrict places that they like to visit. Across the world, innovative housing options, greater transportation opportunities and enhanced outdoor spaces are emerging to better meet the needs and preferences of a growing aging citizenry. Furthermore, mounting research has identified significant health benefits associated with aging and the built environment that is particularly important for persons living increasingly longer lives [1]. In 2006, the World Health Organization (WHO) initiated a broad-scale movement to enhance the age-friendliness of communities by focusing efforts on core features including the built environment [2]. Although the global initiative requires assessing aging residents’ aspirations regarding the built community features, less attention has focused on the correlates of healthy aging and socio-demographic characteristics associated with their views.

This paper will present the findings from a study that examined community-dwelling older adults’ perspectives regarding the built environment (i.e., housing, transportation and outdoor spaces and buildings). This paper begins with an ecological framework of communities and a multi-dimensional model of healthy aging derived from a global public health framework [3]. An overview of the intersection between aging, health and the built environment is provided. Tenets of the WHO effort to enhance the age-friendliness of communities are presented along with a synopsis of the hyper-aged American community under study to provide greater context in understanding the factors associated with the perceived salience of built environmental features according to the older adult participants.

### 1.1. Ecological Context of Healthy Aging

Aging influences and is influenced by the broader environment. Bronfenbrenner’s Theory of Human Ecology identifies a dynamic transactional view of older adults within the context of the built environment on multiple layers including micro (i.e., individuals), meso (i.e., organizations) and macro (i.e., policies and systems) [4]. As people age, many will experience a myriad of changes impacting their functional abilities to manage at home and in the communities in which they reside. In the United States for example, more than one-third (34%) of persons aged 65 to 74 and nearly half of persons (49%) aged 75 and older report one or more types of disability in vision, hearing, cognition, self-care or independent living [5]. The WHO builds upon the ecological theory of aging by explicating that our unique and composite physical and mental capacities, along with the environments we inhabit, comprise the complex interplay of functional ability that enables well-being in older age [3]. The conceptualization of healthy aging therefore comprises physical needs (e.g., mobility status) and mental capabilities (e.g., resilience) and environmental adaptations to enhance functioning in the context of community life (e.g., non-slip flooring). The ecological model further underscores the broader role of supports and services to facilitate aging well in the community.

Beyond the individual, the intricate role between place and health as people age has global implications across society. The WHO explicates a public health framework for healthy aging that identifies a common goal to optimize functional ability across the life course through three stages of capacities (i.e., high, declining and significant loss) [3]. The role of the environment is essential in both the promotion of functional abilities and the removal of barriers for participation in community life in order to compensate for the loss of capacity across varying life-course trajectories. According to the WHO, functional ability “enables older people to do the things they value, including the abilities to meet basic needs, learn, grow and make decisions, be mobile, build and maintain relationships and contribute [3] (pp.19)”. Hence, the notion of healthy aging is an inherently subjective and dynamic sentiment that is uniquely situated in the context of where we live.

### 1.2. Aging, Health and the Built Environment

Mounting research has recognized the role of the physical environment and health among older adults [1]. Housing is a core component of the built environment and is of particular importance to older adults who spend considerably more time in the home setting compared to other age groups [6]. Older Americans overwhelmingly report their desire to age in place and in the communities in which they reside [7]. Aging in place is associated with feelings of affinity towards the home and one’s broader neighborhood as well as improved physical and mental health, functional status and general well-being [8,9]. However, research indicates that many older adults struggle to thrive at home due to a variety of health and other factors [1]. Despite changing needs across the life course, research suggests that the majority of older adults have not considered or implemented universal housing design features that enable the ability to age in place [10].

Transportation provides access to amenities that enable us to remain engaged in our communities. Among the range of mobility options, automobiles provide older adults with a cherished symbol of independence as well as a means of transport [11]. However, age-related and pathological changes in vision, hearing, motor abilities, and cognition impact driving skills and people outlive their ability to drive safely by upwards of ten years [12]. Though urban areas typically have better access to public transportation, demographic characteristics such as female gender, health conditions, lower socio-economic status and built environment features such as neighborhood poverty, street connectivity, and walkability all affect the utilization of public transportation [13]. Failure to access suitable transportation options impacts health via missed medical appointments and leads to increased social isolation [14,15]. Improving accessibility to transportation among older adults is linked to better mental health and social connectivity [16].

There is increasing recognition regarding the health benefits associated with outdoor spaces for older adults [17]. Physical activity in outdoor natural environments is associated with improved physical and mental well-being [18]. Outdoor activities also provide older adults with greater opportunities to socialize thereby countering health concerns associated with isolation and loneliness [19]. Moreover, sidewalk enhancements that improve mobility for persons with assistive devices promotes physical activity among older adults [20]. Although parks are associated with a variety of physical and psychological health benefits, research suggests that neighborhood parks are not well designed for or widely utilized by older adults for physical activities such as walking [21].

### 1.3. Age-Friendly Community Background

The WHO Global Network for Age-Friendly Cities and Communities represents the most broad-scale effort to improve the health of older adults aging in communities [2]. By 2020, more than 1000 communities worldwide have since joined the global movement which began in 2006. Age-friendly communities optimize opportunities for health by addressing eight community features, known as ‘domains of livability’, across the built environment (i.e., housing, transportation, outdoor spaces and public buildings), social environment (i.e., social and civic participation, employment, respect and social inclusion) and service environment (i.e., community support and health services, communication and information) [2]. Participation in the global network requires that communities assess older adults’ input on the eight domains and engage multi-sectors including government to devise and implement actionable steps to improve the age-friendliness of communities.

This study reports on the findings from an assessment of older residents from a mid-sized age-friendly community (approximately 415,000 residents) located in the Southeastern USA. The community is defined by geography and sense of belonging (i.e., identifying as a member of the community). The community is hyper-aged in that nearly half of the residents are aged 60 and older, with a median age of 57 [22]. Notable socio-demographic characteristics of the community include that more than half are White (82%), married (60%), college-educated (65%) and relatively affluent, with income greater than or equal to $50,000 USD (52%) [22]. This study sought to examine the relationship between health and perceptions of the built environment after controlling for potentially influential socio-demographic factors. This was accomplished in two steps: (1) because variability exists in how older adults rate their health and each facet of health (i.e., physical, mental, resiliency) may be correlated with each other, participants were first sorted into homogenous health groups using cluster analysis; (2) once sorted into health clusters, the relationship between health and perceptions of the built environment was examined after controlling for potentially influential socio-demographic factors such as age, gender, education, income, marital status, physical needs, and home adaptations using multiple linear regression.

## 2. Materials and Methods

### 2.1. Study Design and Measures

This study employed surveys to examine older adults’ perceptions of features that are important to enhancing their ability to age in place. A 50 item, self-administered questionnaire was developed based on the WHO checklist of age-friendly domains [2]. Validity of the domain items was derived from the input of more than 2000 older adults and aging experts from 33 cities in 23 countries across the world [2]. The tool addressed socio-demographic data, perceptions regarding built environmental features and multi-dimensional health factors. Because the age-friendly community process builds upon initial assessment data (i.e., survey) in the subsequent construction and implementation of an action plan, the survey questions invoke participants to consider which community features will be the most important to their life and well-being as they age in place. This study represents a subset of a broader study that addressed all eight of the WHO domains of livability, and utilized an expanded analysis to examine correlates associated with the built environmental domains.

Socio-demographic data were measured at the nominal level (i.e., categorically) for the following variables: gender, marital status, education status, income levels and race/ethnicity. Age was measured as a continuous variable.

Perceptions of importance regarding the built environment represented this study’s main dependent variables. The built environment was measured by compiling subscale scores on the perceived importance of three community features: (1) housing included eight items (i.e., ‘well-maintained and safe low-income housing’); (2) transportation included 17 items (i.e., ‘affordable public transportation’); and (3) outdoor spaces and buildings included seven items (i.e., ‘well-maintained and safe parks that are within walking distance of my home’). All facets of the built environment were in response to the participants’ future, “For each item below, please indicate whether you think it will be important to you in the future”, rather than how important they believe the age-friendly amenities to be presently. Subscale scores were determined by summing the ratings of agreement with each item. Reliability of the scales was Cronbach’s α = 0.81 for housing, α = 0.91 for transportation, and α = 0.81 for outdoor spaces and buildings among the full sample.

We applied the WHO definition of healthy ageing to measure dimensions of health by physical, mental, and functional capacities. All items were measured by self-report on an ordinal scale. Self-reported physical health ranged from “poor: 1” to “excellent: 5” on a question, “In general, when compared to most people your age, how would you rate your health?” Self-reported mental health ranged from “poor: 1” to “excellent: 5” on a question, “How would you rate your overall mental/emotional well-being?” We developed a resiliency sum score to capture the psychological dimension of functional capacity by creating a summed score of four items, with scores ranging from 0 to 4 (i.e., ‘I am able to make choices about things that affect how I age’, ‘I am able to adjust to changes that are related to aging’, ‘I am able to cope with the challenges of my later years’, and ‘I am able to meet all of my needs and some of my wants’). Social connectedness to others was estimated with a yes/no question, “I feel connected to other people”.

To further operationalize the construct of functional capacities, we summed a total of physical needs ranging from 0 to 6 based on use of canes, eyeglasses, hearing aids, walkers, wheelchairs, and use of special transportation services such as one for seniors or people with disabilities. As an element of environmental fit, we utilized a summed score of home adaptations ranging from 0 to 6, including ‘Easier access into or within your home such as a ramp, chairlift, or elevator or wider doorway’, ‘Bathroom modifications such as grab bars, handrails, high toilet or non-slip tile’, ‘Putting a bedroom, bathroom and kitchen on the first floor’, ‘Improving lighting’, ‘Installing a medical emergency response system that notifies others in case of emergency’, and ‘Non-slip floor surfaces’.

### 2.2. Sampling

We utilized a purposive design targeting persons aged 50 and older that were residents of the community. As representatives of the young-old and soon-to-be older adult age group (i.e., age 65+), we deliberately included the Baby Boomer generation, who were aged 50 to 69 at the time of this study. The survey was available in both English and Spanish as well as print and electronic versions, widely promoted via traditional and social media as well as places with high older adult traffic throughout the community. Deliberate efforts to promote the survey were conducted across the care continuum to include input from older adults residing independently in the community as well as those residing in senior housing.

### 2.3. Process and Procedures

The research received Institutional Review Board approval (eIRB # 00020938) from the University. Informed consent was obtained prior to participation. The survey was available in print or electronically by participants at their own pace, and took approximately 20 min to complete. Instructions to facilitate anticipation on the perceived importance of community features were provided in the cover letter accompanying the survey.

### 2.4. Participants and Exclusion Criteria

A total of 1127 persons participated in this study. Given that missingness was deemed missing not at random (i.e., respondents chose which questions to skip), analyses were conducted after excluding all missingness on the variables of interest, including the built environment sum scores, self-rated measures, and demographics. This led to a final sample of 598 older adults (*n* = 426 excluded due to missingness on the built environment sum scores, *n* = 103 excluded due to missingness on demographics and self-reported measures). Cronbach’s α for the built environment sum scores was similar for the smaller sample with no missing data (α = 0.80 for housing, α = 0.91 for transportation, and α = 0.80 for outdoor spaces and buildings).

### 2.5. Data Analysis

In order to extract homogenous subgroups from the heterogenous sample of community-dwelling older adults, we utilized K-means cluster analysis. Self-reported physical health, self-reported mental health, and the resiliency sum score were first converted to Z-scores (i.e., mean of zero, standard deviation of one). An algorithm was developed to plot the within-groups sum of squares by the number of potential clusters in order to identify the ideal number of clusters. The ideal number of clusters was determined to be four in our sample. (See Supplementary Figure S1).

Analysis of variance and chi-square tests were used to compare clusters on a variety of health and demographic measures. When the chi-square statistic was difficult to estimate due to sparseness of cells in variables with many categories (e.g., marital status, education, income), a Monte Carlo-simulated *p*-value from 2000 replicates was reported to reduce the risk of type I or type II errors. Finally, linear regression was used to examine the relationship between health clusters and built environment perspectives. Cluster 3 was used as the reference category because it was the largest and was easiest to compare to the other three clusters (i.e., Cluster 3 rated all facets of health as high). Covariates included age, gender (‘Female’ as the reference category), race (‘non-White race as the reference category), marital status (‘not married’ as the reference category), education as an ordinal variable (‘1′ K-12 to ‘6′ Graduate School), income as an ordinal variable (‘1′ < $10,000 to ‘8′ > $150,000), number of physical needs (0 to 6), and home adaptations (0 to 6). Survey data were analyzed using R 3.6.1 (RStudio, PBC, Boston, MA, USA).

## 3. Results

### 3.1. Clusters of Community-Dwelling Older Adults by Health

**Figure 1:**
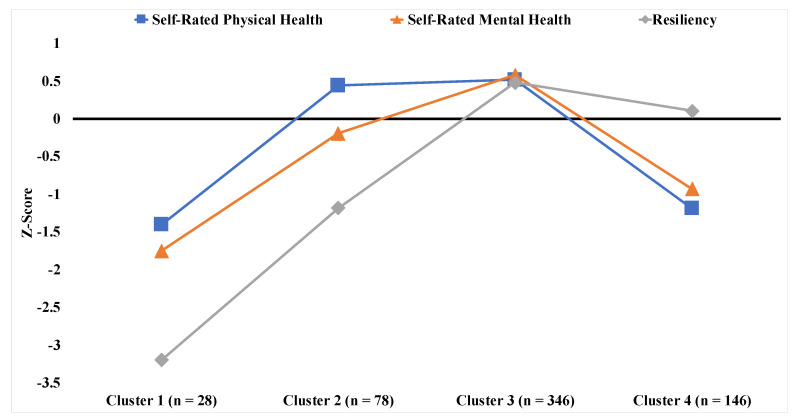
K-means cluster analysis on self-rated physical health, self-rated mental health, and resiliency. Note. *n* = 598. Higher Z-scores represent better health, mental health, or resiliency.

Four clusters were extracted from the K-means cluster analysis based on the 598 participants with full self-report and demographics data. Figure 1 provides a graphical representation of each cluster’s reporting by self-reported physical health, mental health and resiliency. Cluster 1 (*n* = 28) was characterized by poor physical health, poor mental health, and poor resiliency. Cluster 2 (*n* = 78) was characterized by above-average physical health, average mental health, and poor resiliency. Cluster 3 (*n* = 346) was characterized by excellent physical health, excellent mental health, and excellent resiliency. Cluster 4 (*n* = 146) was characterized by average resiliency, but poor physical health and mental health.

Table 1 identifies characteristics of each cluster by built environmental ratings, health dimensions, functional capacities and socio-demographic data. The lowest rating on social connectedness was found among Cluster 1, of which only 57% reported feeling connected to others, compared to 82% of Cluster 4, 86% of Cluster 2, and 97% of Cluster 3 (*p* < 0.001). On functional capacities, the clusters differed on the number of physical needs (*p* = 0.002), with Clusters 1 and 4 having the greatest needs and Cluster 2 having the fewest. While participants in each cluster reported at least some home adaptations to age in-place, the number of home adaptations was highest in Clusters 1 and 4 and lowest in Cluster 2 (*p* = 0.004).

The clusters significantly differed according to several socio-demographics. Age varied by cluster (*p* < 0.001), with Cluster 1 being the youngest and Cluster 3 being the oldest. While Clusters 1, 2, and 4 were relatively similar on the proportion identifying as male, Cluster 3 was more often male (41% vs. 23–29%; *p* = 0.002). Education (*p* = 0.002) and income (*p* < 0.001) also differed significantly with greater educational attainment and wealth identified in Cluster 3 and the least educated and wealthy noted in Cluster 1. For example, 91% of Cluster 3 had a college degree compared to 79% of Cluster 1, and 82% of Cluster 3 reported annual earnings of $50,000 USD or greater compared to just 50% of Cluster 1.

### 3.2. Perspectives on the Built Environment

As noted in Table 2, there were no significant differences found on older adult participants’ views on outdoor spaces and buildings by cluster or after controlling for socio-demographic variables and functional capacities (i.e., age, gender, race, marital status, education, income, number of physical needs and home adaptations). However, age was negatively associated with older participants’ ratings on the perceived importance of outdoor spaces and buildings (*p* = 0.01).

There were significant differences between clusters on housing (*p* = 0.008). Cluster 3 reported the least importance on housing whereas Cluster 1 reported the most importance. However, only Cluster 4 reported a higher importance of housing in comparison to Cluster 3 after controlling for covariates (*p* = 0.04). While a greater number of home adaptations was positively associated with the importance of housing (*p* = 0.007), increasing age was found to be negatively associated with the importance of housing (*p* < 0.001).

There were significant differences in transportation between clusters (*p* < 0.001). Similar to housing, Cluster 3 reported the least importance on transportation, whereas Cluster 1 reported the most importance. After controlling for socio-demographic variables and functional capacities, Clusters 1, 2, and 4 reported greater interest in transportation in comparison to Cluster 3 (Cluster 1 *p* = 0.03; Cluster 2: *p* = 0.01; Cluster 4: *p* = 0.03). Additionally, a higher income was negatively associated with viewing transportation as important in the future (*p* = 0.02), while more home adaptations to age in place was positively associated with increased interest in transportation features (*p* = 0.001).

## 4. Discussion

This study sought to determine community-dwelling older adults’ preferences on built environmental features as it related to multiple health dimensions, functional capacities and socio-demographic variables. Using cluster analysis, we identified four unique clusters of which two were most distinct in representing the opposite spectrums of health (i.e., Cluster 1 was characterized by poor physical health, mental health, and resiliency compared to Cluster 3, characterized by excellent physical health, mental health, and resiliency). The range of characteristics associated with the clusters underscores the diversity of community-dwelling older adults and the wide range of factors associated with views on built environmental features. Our findings support previous research regarding the nuanced relationship between self-rated health, satisfaction on built environmental features and socio-demographic characteristics [23]. However, the regression models only explained 1% to 7% of the variability in built environment preferences. This finding highlights the importance of engaging older adults to understand their preferences on built environmental features in age-friendly communities rather than making assumptions based on socio-demographics or health status. Indeed, researchers and public policy advocates must recognize the heterogeneity of individual circumstances in community-dwelling older adults when developing and disseminating age-friendly communal characteristics [24].

After controlling for potentially influential covariates, we found that the health cluster was not a significant factor when estimating self-rated importance of outdoor spaces and buildings. Our findings reinforce previous research that identified younger age as associated with greater utilization of outdoor spaces as we also found older age was associated with lower importance of age-friendly outdoor spaces and buildings [25]. It should be noted that this finding was consistent after controlling for health and disability-related factors, potentially suggesting cohort differences or other unmeasured factors that should be investigated in the future. Since the dissemination of the Americans with Disabilities Act, buildings across the USA have improved their accessibility status dramatically. However, barriers to inclusion still exist in public outdoor spaces, both within urban and rural settings. Public health efforts can incorporate explicit attention to advanced age vis-à-vis the promotion of health and removal of barriers that facilitate outdoor activity for persons across all ages and abilities [26].

Our study identified housing as most important by those with poor physical health, poor mental health, and poor to average resiliency per Clusters 3 and 4. However once again, older age was associated with lower self-rated importance of age-friendly housing. It is unknown whether the older participants in the age-friendly community are truly content with their current housing, resigned themselves to accepting where they reside or were simply unaware of options to age better at home among a host of other alternative reasons. However in part buttressing that age-friendly housing features are indeed important to one’s future, participants in this study that reported more home adaptations to age in place at home placed a greater importance on housing. Greater attention to educating older adults, families, government and businesses on ways to enhance functioning via simple home adaptations may be particularly helpful to promote well-being at home. For example, age-friendly communities can promote the awareness and adaption of low cost universal design provisions, such as improved lighting and flooring.

On the importance of transportation, participants comprising Cluster 1, reporting the worst health of all, rated age-friendly transportation as more important to their future, while participants comprising Cluster 3, with the best overall health, reported less importance on transportation. Furthermore, income was negatively associated with the importance of transportation, suggesting that healthier and more affluent older adults may not perceive future mobility needs. Those reporting more home adaptations also placed a greater importance on transportation. Combined, these findings suggest that public health efforts could differentially target education and interventions regarding transportation that recognizes perceptions of vulnerabilities by health, home and socio-demographics such as age and income. For example, older people with subjectively good health and economic resources, who have already adapted their home, may simply not foresee a future of declining driving abilities, yet might respond to an appealing message to help better prepare for future mobility. Emergent transport options such as ride sharing, autonomous vehicles and home-delivered services can also be promoted to improve access for those no longer able to drive and in cases or locales where public transportation is neither preferred or available.

Beyond the public health practice implications aforementioned, findings from this study pose further considerations for theory, policy and research as well. For example, our findings denoting an increased importance on housing and transportation found among those with most home adaptations bolster empirical support for both Bronfenbrenner’s ecological theory and the WHO’s conceptualization of healthy ageing [3,4], which recognizes a transactional relationship between older adults adapting to their environment. In addition, study findings indicating that those with higher incomes placed less importance on features such as transportation revealed that wealthy individuals may not recognize or value transportation infrastructure across the broader community. Municipal mobility efforts, particularly in times of austerity post-COVID-19, may need to be crafted to address the utility of public expenditures in roadways, sidewalks and other forms of travel as investments and economic savings for communities in order to garner buy in from an already divided constituency. Framing of transportation policies that explicitly address persons of all ages may be particularly useful as well. The inverse relationship we found on increased age and decreased importance on both outdoor spaces and housing is not well understood and should be further studied. For example, previous research suggests that older adults place a greater emphasis on aging in place [6,7,8]. Qualitative inquiry via interviews, observation or case study could reveal important insight regarding how older adults manage or face barriers in these settings.

Our findings are not without limitations. This study was conducted in a largely homogenous age-friendly community in the United States, whose respondents and the broader built environment may substantially differ elsewhere. In addition, the cluster and regression analyses provided were observational and do not provide casual inferences. Though the built environment sum scores had good internal reliability, they only represent a cross-section of the numerous features available in a truly age-friendly community. It is conceivable that some participants who were excluded due to missingness were different from those included in our sample. Participants included were younger, more likely to be White, more likely to be married, and had a higher income in comparison to those excluded. However, there were no differences in the main independent variables (i.e., self-rated health, self-rated mental health, or resiliency). Future work should examine whether our findings hold in more diverse samples, including adults in the oldest-old category of life (85 years or older), racial and ethnic minorities, unmarried older adults, and those of lower socio-economic status. In comparison to the overall sample, our smaller sample size certainly affected the statistical power. Moreover, loss of power may have been exacerbated by the small sample sizes of some of the clusters (e.g., especially Clusters 1 and 2 in comparison to Clusters 3 and 4). This may have unintentionally led to type II errors when comparing the smaller clusters to the largest (Cluster 3: *n* = 346).

## 5. Conclusions

This study examined older adults’ perspectives on the built environment by correlates of healthy aging in an age-friendly community. Cluster and regression analytic techniques were conducted and four distinct profiles were identified, with the greatest preference for housing and transportation found among those reporting poorer health compared to those reporting excellent health across multi-dimensional healthy aging measures. Perceptions on the importance of built environmental features were also found to vary by age, income and home accessibility status. Findings underscore the need for public health professionals to directly assess the perspectives of their community members on the built environment, as the majority of the variability in responses could not be explained by socio-demographic and health features.

## Figures and Tables

**Table 1:** Characteristics of community-dwelling older adults (*n* = 598) clustered by built environmental domains, heath dimensions, functional capacities and socio-demographics.

	Cluster 1 *N* = 28	Cluster 2 *N* = 78	Cluster 3 *N* = 346	Cluster 4 *N* = 146	*p*
	*M* (SD)% (*n*)	*M* (SD)% (*n*)	*M* (SD)% (*n*)	*M* (SD)% (*n*)	
Built Environment Domains					
Outdoor Spaces and Buildings	5.82 (1.61)	5.78 (1.60)	5.87 (1.66)	5.78 (1.74)	0.94
Housing	6.36 (1.68)	6.21 (2.12)	5.61 (2.21)	6.20 (2.09)	0.008
Transportation	15.86 (1.92)	15.14 (3.31)	13.64 (4.27)	14.86 (3.43)	<0.001
Health Dimensions					
Self-Rated Physical Health	2.71 (0.66)	4.37 (0.51)	4.44 (0.54)	2.90 (0.59)	<0.001
Self-Rated Mental Health	2.54 (0.79)	3.92 (0.73)	4.61 (0.49)	3.27 (0.69)	<0.001
Social Connectedness (Yes)	57% (16)	86% (67)	97% (336)	82% (120)	<0.001
Functional Capacities					
Resiliency	1.25 (0.84)	2.76 (0.49)	4.00 (0.00)	3.72 (0.45)	<0.001
Physical Needs	1.21 (1.03)	0.87 (0.69)	1.01 (0.65)	1.21 (0.79)	0.002
Home Adaptations	1.46 (1.55)	0.74 (1.18)	1.09 (1.40)	1.40 (1.42)	0.004
Demographic Covariates					
Age	62.86 (9.01)	65.73 (9.20)	68.76 (8.70)	67.58 (9.08)	<0.001
Gender (% Male)	29% (8)	23% (18)	41% (142)	27% (39)	0.002
Race (% Non-White Race)	0% (0)	5% (4)	5% (17)	4% (6)	0.67
Marital Status					0.07
Married	61% (17)	63% (49)	64% (220)	63% (92)	
Divorced	14% (4)	22% (17)	14% (49)	12% (18)	
Widowed	7% (2)	6% (5)	13% (44)	13% (19)	
Never Married	14% (4)	9% (7)	3% (12)	5% (8)	
Unmarried and Cohabiting	4% (1)	0% (0)	6% (21)	6% (9)	
Education					0.002
K-12th Grade	7% (2)	4% (3)	0% (0)	0% (0)	
High School Graduate	4% (1)	1% (1)	2% (6)	3% (4)	
Post-High School Education	11% (3)	9% (7)	7% (25)	15% (22)	
2 year College Degree	14% (4)	13% (10)	9% (30)	11% (16)	
4 year College Degree	36% (10)	27% (21)	35% (120)	32% (47)	
Graduate/Profess Degree	29% (8)	46% (36)	48% (165)	39% (57)	
Income					<0.001
<$10,000	14% (4)	3% (2)	1% (5)	2% (3)	
$10,000–$19,999	4% (1)	4% (3)	1% (4)	5% (8)	
$20,000–$29,999	11% (3)	14% (11)	4% (15)	5% (7)	
$30,000–$49,999	21% (6)	10% (8)	11% (39)	23% (33)	
$50,000–$74,999	11% (3)	22% (17)	20% (68)	21% (30)	
$75,000–$99,999	7% (2)	15% (12)	16% (57)	21% (31)	
$100,000–$149,000	7% (2)	26% (20)	16% (54)	12% (17)	
>$150,000	25% (7)	6% (5)	30% (104)	12% (17)	

Note. ANOVAs and chi-square tests were used to extract *p*-values. Where the chi-square statistic was difficult to estimate due to small cell numbers a simulated *p*-value based on 2000 replicates was used. Physical needs range from 0 to 6. Home adaptations range from 0 to 6.

**Table 2:** Linear regression comparing Clusters 1, 2, and 4 to Cluster 3 on outdoor spaces and buildings, housing, and transportation after controlling for functional capacity and socio-demographics.

	Outdoor Spaces and Buildings		Housing		Transportation	
	Beta [95% CI]	*p*	Beta [95% CI]	*p*	Beta [95% CI]	*p*
Cluster 1 (ref = 3)	−0.14 [−0.80, 0.53]	0.68	0.38 [−0.46, 1.22]	0.37	1.71 [0.17, 3.25]	0.03 *
Cluster 2 (ref = 3)	−0.18 [−0.60, 0.24]	0.40	0.42 [−0.11, 0.95]	0.12	1.29 [0.31, 2.26]	0.01 *
Cluster 4 (ref = 3)	−0.13 [−0.47, 0.21]	0.46	0.44 [0.02, 0.87]	0.04 *	0.87 [0.09, 1.66]	0.03 *
Age	−0.02 [−0.04, 0.00]	0.01 *	−0.05 [−0.07, −0.03]	<0.001 ***	−0.03 [−0.07, 0.01]	0.11
Gender (Male)	−0.12 [−0.42, 0.18]	0.44	−0.05 [−0.43, 0.33]	0.80	−0.41 [−1.11, 0.28]	0.24
Race (White)	−0.66 [−1.31, −0.01]	0.05	−0.42 [−1.23, 0.39]	0.31	0.17 [−1.33, 1.66]	0.83
Marital Status (Married)	0.17 [−0.16, 0.50]	0.31	0.34 [−0.07, 0.75]	0.11	0.20 [−0.55, 0.96]	0.60
Education	0.07 [−0.06, 0.20]	0.28	0.11 [−0.06, 0.27]	0.21	0.25 [−0.05, 0.55]	0.10
Income	−0.03 [−0.13, 0.06]	0.48	−0.10 [−0.21, 0.02]	0.10	−0.27 [−0.48, −0.05]	0.02 *
Physical Needs	−0.09 [−0.29, 0.12]	0.40	−0.12 [−0.38, 0.13]	0.34	−0.12 [−0.59, 0.35]	0.62
Home Adaptations	0.04 [−0.06, 0.14]	0.42	0.17 [0.05, 0.30]	0.007 **	0.38 [0.14, 0.61]	0.001 **

Note. * *p* < 0.05, ** *p* < 0.01, *** *p* < 0.001. CI = confidence interval. Education ranges from 1 to 6 (K-12 to Graduate School). Income ranges from 1 to 8 (<$10,000 to >$150,000). Physical needs range from 0 to 6. Home adaptations range from 0 to 6. Adjusted-R^2^ outdoor spaces and buildings: 1%; adjusted-R^2^ housing: 7%; adjusted-R^2^ transportation: 5%.

## Supplementary Materials

The following are available online at https://www.mdpi.com/1660-4601/17/19/7056/s1. Figure S1: Within groups sum of squares by the number of clusters for K-means cluster analysis.
