# Supplementary material for: Examining Older Adults’ Perspectives on the Built Environment and Correlates of Healthy Aging in an American Age-Friendly Community
Source: Int J Environ Res Public Health. 2020 Sep 27;17(19):7056. doi: 10.3390/ijerph17197056 (PMC7578930; doi:10.3390/ijerph17197056)
Supplement: Supplementary file 1 [file ijerph-17-07056-s001.pdf]

Supplementary Material: Supplementary Figure S1.

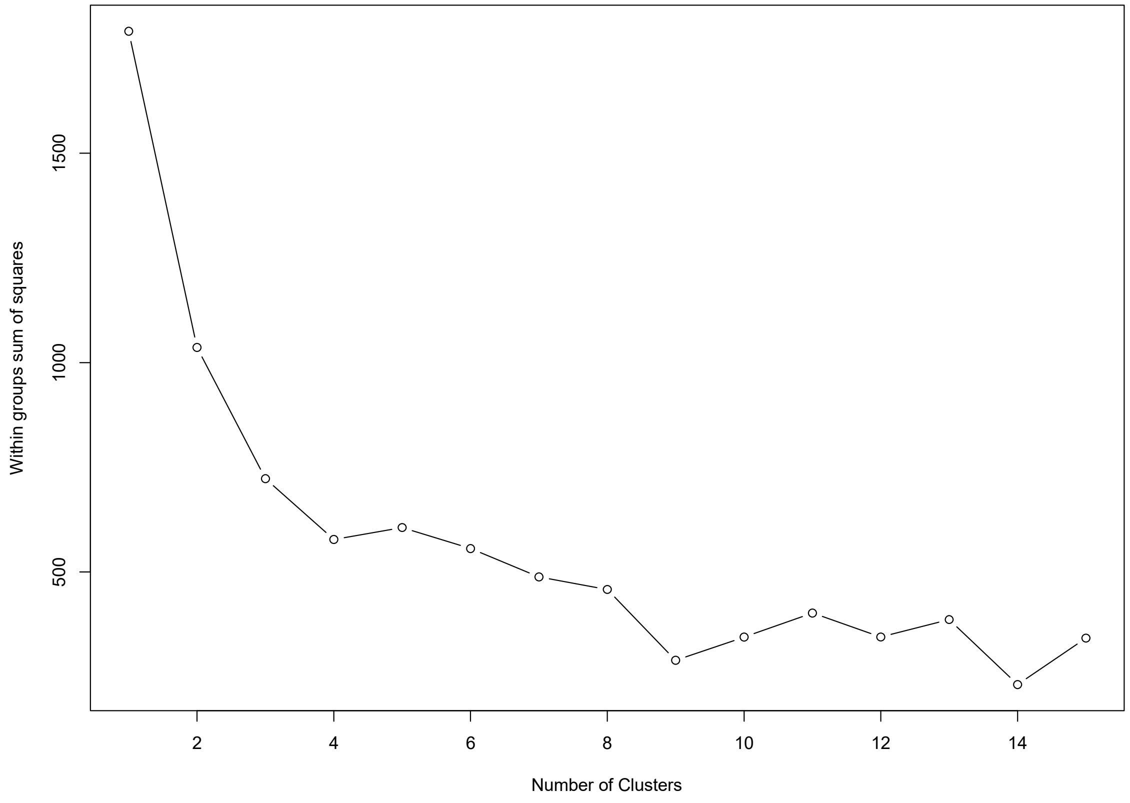

Supplementary Figure S1. Within groups sum of squares by the number of clusters for K-means cluster analysis.
